# Intraductal Papillary Neoplasm With Associated Invasive Cholangiocarcinoma and Biliary Intraepithelial Neoplasia in Primary Sclerosing Cholangitis: A Case Report

**DOI:** 10.7759/cureus.105924

**Published:** 2026-03-26

**Authors:** Ziad Letaïef, Kamal Fawaz Nijim, Mohammed Jasim Al-Janabi

**Affiliations:** 1 Radiology, UZ Brussel, Jette, BEL

**Keywords:** biliary intraepithelial neoplasia, intraductal papillary mucinous neoplasm of the bile duct (ipmn-b), intrahepatic cholangiocarcinoma, magnetic resonance cholangiopancreatography (mrcp), primary sclerosing cholangitis (psc)

## Abstract

Intraductal papillary neoplasm of the bile duct (IPNB) is a biliary epithelial tumor that represents a recognized precursor lesion of cholangiocarcinoma. We report the case of a 60-year-old woman who presented to the emergency department with acute abdominal pain. A CT scan performed for suspected nephrolithiasis incidentally revealed an irregular hypodense lesion in segment VI of the liver with associated intrahepatic bile duct dilatation. Further evaluation with MRI and magnetic resonance cholangiopancreatography (MRCP) demonstrated a papillary intraductal mass within a dilated bile duct, appearing hyperintense on T2-weighted images and hypointense on T1-weighted images, and showing diffusion restriction and early contrast enhancement. Based on these findings, a preliminary diagnosis of IPNB was made, and the patient underwent right hepatic resection. Histopathological examination confirmed IPNB of mixed intestinal and pancreatobiliary type with high-grade dysplasia, associated with two foci of invasive cholangiocarcinoma. Additional findings included biliary intraepithelial neoplasia and primary sclerosing cholangitis with advanced fibrosis. This case highlights the importance of recognizing the imaging features of intraductal biliary tumors and demonstrates the value of MRI with MRCP in establishing the diagnosis. Early detection of IPNB is crucial, particularly in patients with underlying inflammatory biliary disease, as timely surgical treatment may prevent progression to advanced cholangiocarcinoma.

## Introduction

Intraductal papillary neoplasm of the bile duct (IPNB) is a preinvasive biliary epithelial tumor characterized by papillary proliferation of epithelial cells supported by fibrovascular cores within the bile duct lumen. Based on the degree of cytoarchitectural atypia of the lining epithelium, IPNBs are histopathologically categorized as lesions with low- or intermediate-grade intraepithelial neoplasia and those with high-grade intraepithelial neoplasia [1]. IPNB is widely considered the biliary counterpart of pancreatic intraductal papillary mucinous neoplasms (IPMNs), as both entities share similar morphological characteristics, including papillary intraductal growth and frequent mucin production, which is reported in approximately 30%-40% of cases [2]. These lesions follow a comparable stepwise progression toward malignancy, and therefore, the same histopathological grading system has been adopted for IPNB [3]. Importantly, invasive cholangiocarcinoma is already present in a substantial proportion of cases at diagnosis (40%-80%). For this reason, the World Health Organization (WHO) classification also includes a third category, IPNB with associated invasive carcinoma, analogous to the classification used for IPMN [2,4].

From a histological perspective, IPNBs can be further divided into four epithelial subtypes: pancreatobiliary, intestinal, gastric, and oncocytic. Mixed phenotypes are not uncommon [5,6]. More recently, Japanese and Korean pathologists proposed an additional two-tier classification system (type 1 and type 2) that correlates with these epithelial subtypes. Type 1 lesions are typically found in the intrahepatic bile ducts and are often associated with mucin production and lower-grade dysplasia, whereas type 2 lesions more frequently arise in the extrahepatic bile ducts and tend to demonstrate high-grade dysplasia [7]. In Western populations, the pancreatobiliary subtype is most frequently encountered and is commonly associated with high-grade dysplasia and invasive carcinoma. In contrast, the intestinal subtype is more prevalent in Asian countries and is typically characterized by more abundant mucin secretion [8]. IPNB may arise anywhere along the biliary tree and can therefore be classified according to its anatomical location as intrahepatic IPNB, extrahepatic IPNB, combined forms, or lesions originating from the epithelium of the peribiliary glands. Intrahepatic lesions are more frequently reported in Asian populations and often occur in the left hepatic lobe, whereas extrahepatic forms are more commonly observed in Western countries [2].

## Case presentation

A 60-year-old woman presented to the emergency department with acute abdominal pain that started suddenly at night. The pain was diffuse but predominantly localized to the right side of the abdomen and was described as constant and pressure-like, with intermittent stabbing exacerbations. She reported one episode of vomiting earlier that day and subsequent anorexia. Bowel movements were preserved without diarrhea or hematochezia. The patient also reported chills and subjective fever but no objectively measured fever. She denied urinary symptoms, although she had a known history of nephrolithiasis and cholelithiasis.

Her past medical history included ulcerative colitis, metabolic dysfunction-associated steatotic liver disease (previously diagnosed as non-alcoholic steatohepatitis), and prior sleeve gastrectomy. For completeness, the patient’s current medications included semaglutide, venlafaxine, lisdexamfetamine, and intermittent lorazepam.

On admission, vital signs were stable with a temperature of 36.1°C, oxygen saturation of 97%, pulse of 86 beats/min, and blood pressure of 134/122 mmHg. Physical examination revealed a soft abdomen with marked tenderness predominantly in the right lower quadrant and right flank. Mild epigastric and left lower quadrant tenderness was also noted. Cardiopulmonary examination was unremarkable, and the patient was neurologically oriented.

Laboratory investigations showed mild leukocytosis (11.7 × 10³/mm³) with neutrophilia, while C-reactive protein was negative and renal function was normal. In addition, liver function tests demonstrated biochemical signs of cholestasis, with elevated alkaline phosphatase (494 U/L), gamma-glutamyl transferase (260 U/L), aspartate aminotransferase (243 U/L), and alanine aminotransferase (229 U/L). Urinalysis demonstrated significant hematuria without pyuria.

**Figure 1:**
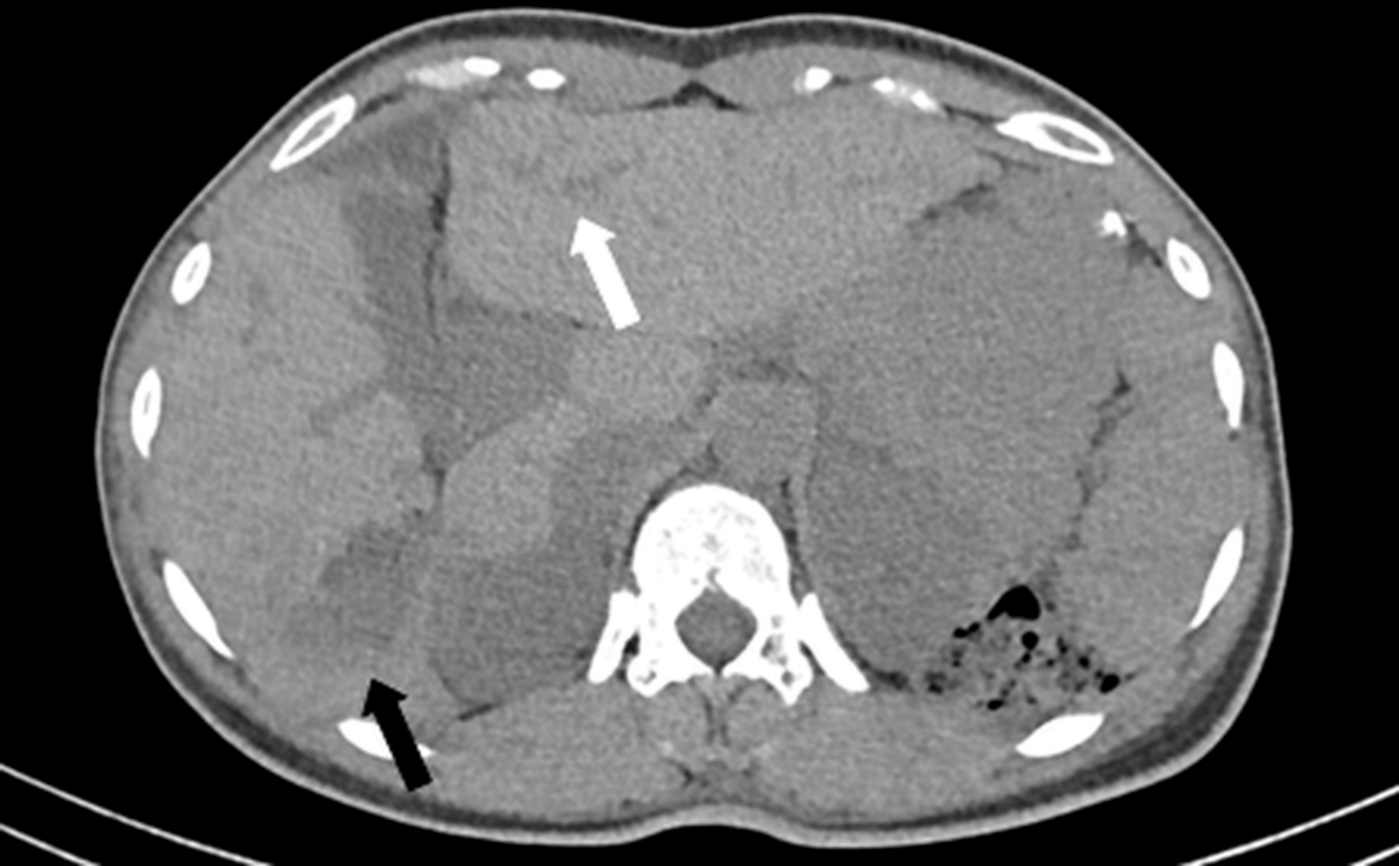
Axial non-enhanced CT image at the level of the liver Axial non-enhanced CT image at the level of the liver demonstrating a globular lesion in segment VI that appears relatively hypodense compared with the surrounding liver parenchyma and measures 4.5 cm x 2.7 cm. The lesion shows heterogeneous attenuation with areas of relatively higher density (black arrow). Irregular dilatation of the intrahepatic bile ducts is also visible (white arrow).

A CT scan performed to evaluate suspected nephrolithiasis demonstrated a non-obstructive 5-mm calculus in the right kidney without hydronephrosis. In addition, cholecystolithiasis was identified without imaging features of acute cholecystitis. Incidentally, a 4.5 cm × 2.7 cm irregular, hypodense lesion with ill-defined margins was detected in the posterior segment of the right hepatic lobe (segment VI) (Figure 1). The lesion demonstrated heterogeneous low attenuation relative to the surrounding liver parenchyma, without evidence of calcification or intralesional hemorrhage. Diffuse intrahepatic bile duct dilatation was present, more pronounced in the segments adjacent to the lesion, with regions of marked dilatation alternating with areas of mild dilatation and focal strictures.

**Figure 2:**
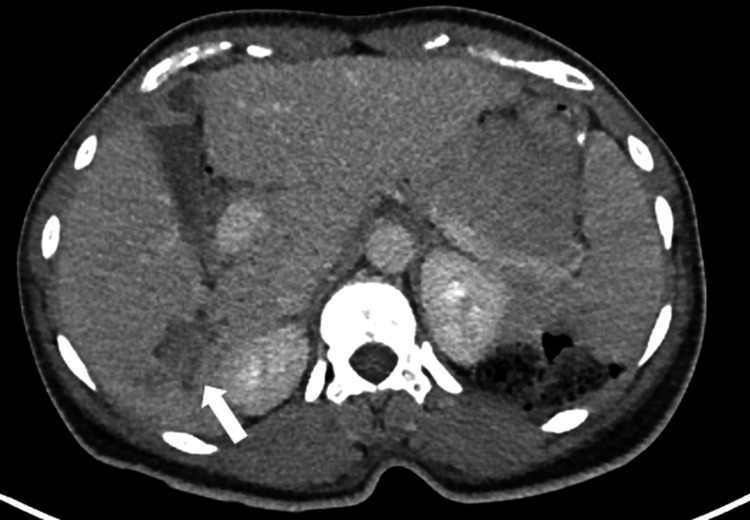
Axial contrast-enhanced CT image of the liver in the portal venous phase Axial contrast-enhanced CT image in the portal venous phase showing the lesion with enhancement similar to the surrounding liver parenchyma (white arrow).

**Figure 3:**
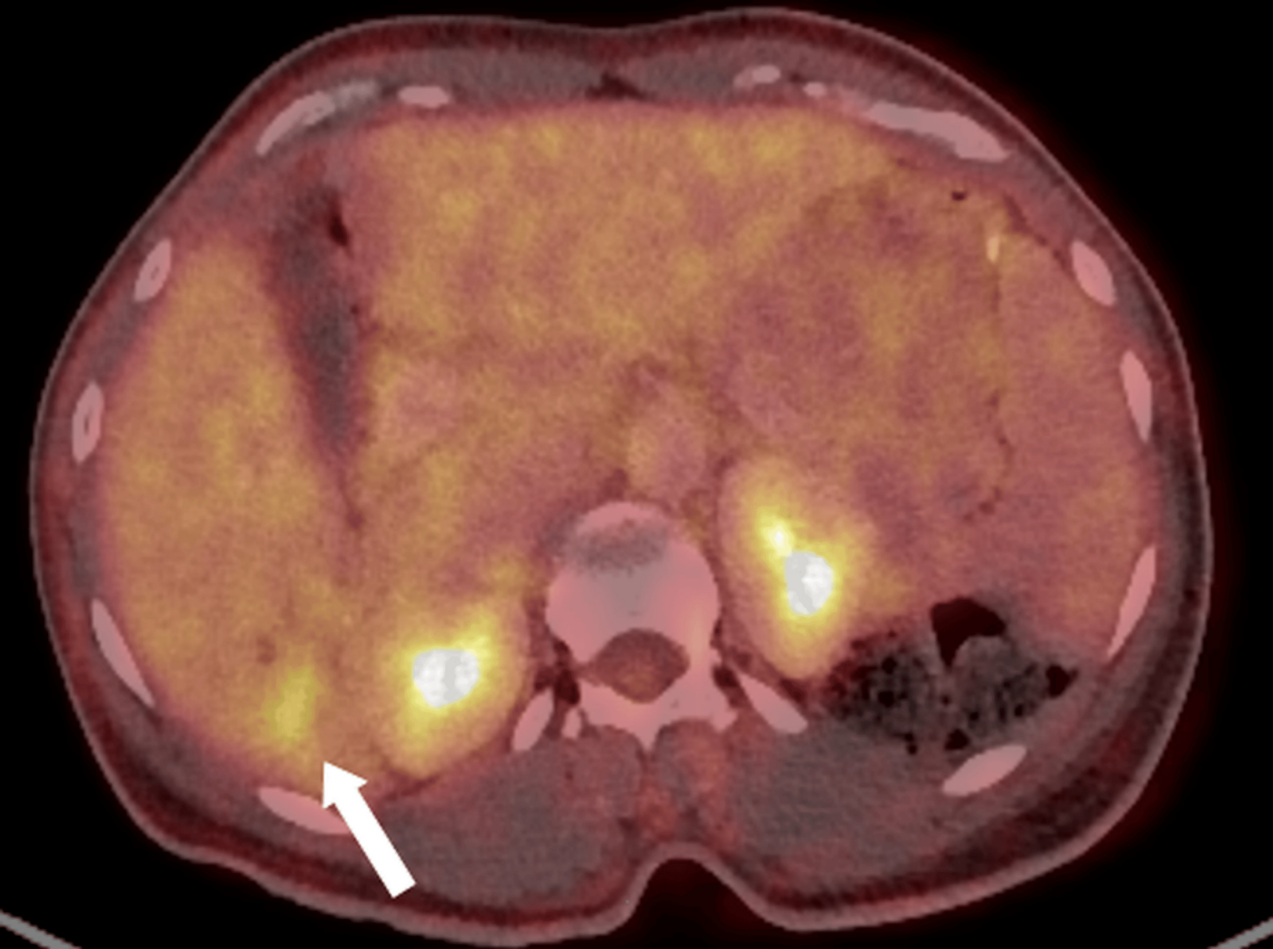
Axial PET-CT image of the liver Axial PET-CT image demonstrating mild hypermetabolic activity within the hepatic lesion in segment VI (white arrow).

On contrast-enhanced CT, part of the lesion showed enhancement similar to the surrounding liver parenchyma during the portal venous phase, compatible with a solid component, while other areas remained hypodense, consistent with bile duct dilatation secondary to the lesion (Figure 2). There was no clear arterial phase hyperenhancement or washout. Subsequent PET-CT demonstrated mild hypermetabolic activity within the lesion (Figure 3). Given the patient’s history of ulcerative colitis and metabolic liver disease, further hepatobiliary evaluation with MRI and magnetic resonance cholangiopancreatography (MRCP) was performed to better characterize the lesion and exclude hepatocellular carcinoma.

**Figure 4:**
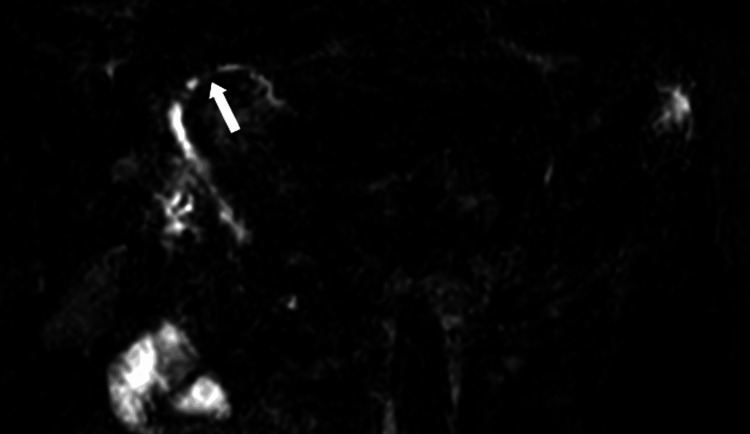
MRCP image of the liver MRCP image demonstrating irregular intrahepatic bile duct dilatation with multifocal strictures, consistent with an abnormal biliary tree configuration (white arrow). MRCP, magnetic resonance cholangiopancreatography.

**Figure 5:**
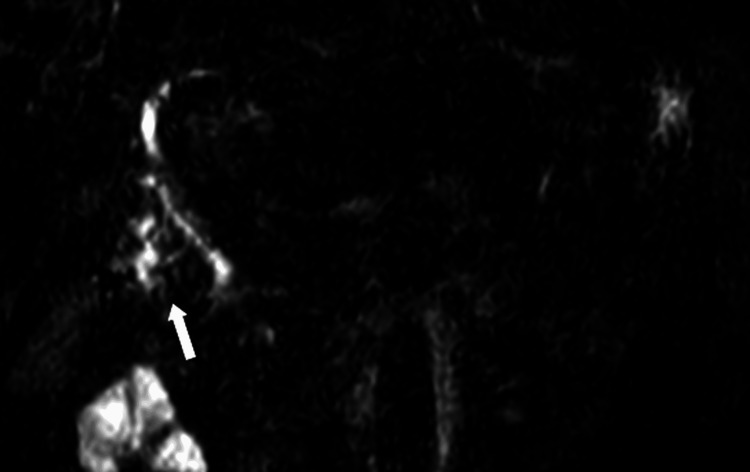
MRCP image of the liver depicting the lesion MRCP image showing marked dilatation of the bile duct in segment VI corresponding to the location of the lesion previously identified on CT (white arrow). MRCP, magnetic resonance cholangiopancreatography.

**Figure 6:**
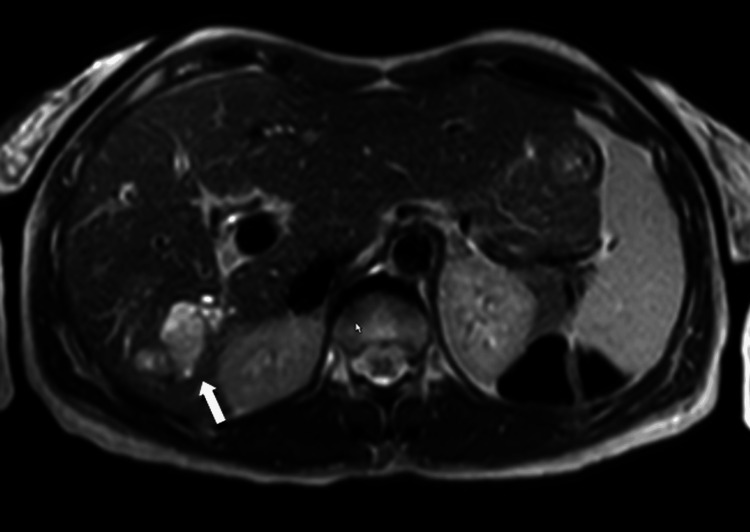
Axial T2-weighted MRI image at the level of the lesion Axial T2-weighted MRI demonstrating a papillary intraductal mass within a dilated bile duct in segment VI, appearing slightly hyperintense compared with the surrounding liver parenchyma (white arrow).

**Figure 7:**
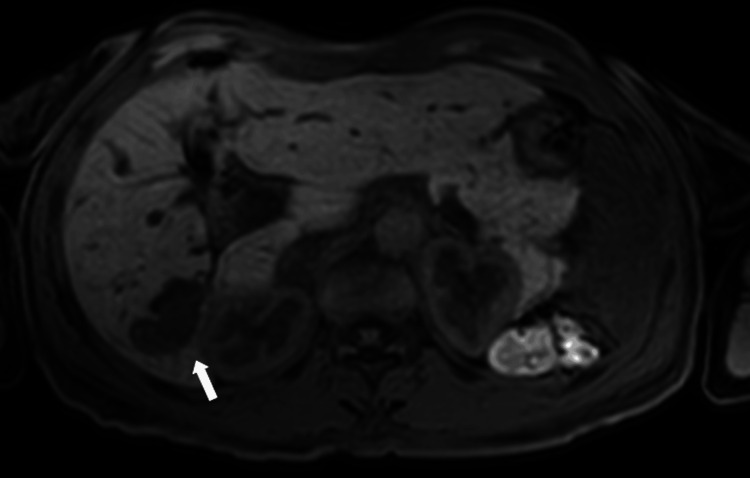
Axial T1-weighted MRI image at the level of the lesion Axial T1-weighted MRI showing the same intraductal lesion appearing hypointense relative to the liver parenchyma (white arrow).

MRI with MRCP was subsequently performed for further characterization of the lesion and correlation with prior CT findings. MRCP (Figure 4) demonstrated irregular intrahepatic bile duct dilatation with multiple strictures, resulting in a beaded appearance suggestive of an underlying sclerosing cholangiopathy such as primary sclerosing cholangitis (PSC), with the most pronounced dilatation in segment VI corresponding to the lesion previously identified on CT (Figure 5). On T2-weighted images (Figure 6), the lesion appeared as a papillary intraductal growth within a dilated bile duct, corresponding histologically to papillary epithelial proliferation as confirmed on resection specimen. On T1-weighted images (Figure 7), the lesion was hypointense.

**Figure 8:**
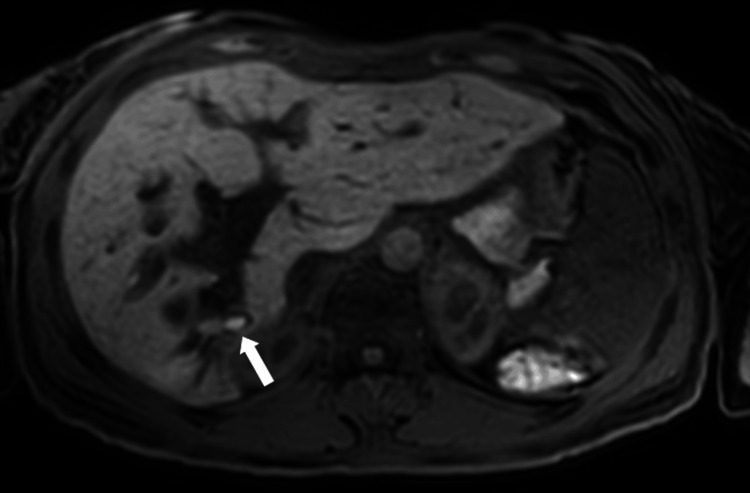
Axial T1-weighted MRI of the liver Axial T1-weighted MRI demonstrating biliary casts within the bile ducts, appearing hyperintense (white arrow).

**Figure 9:**
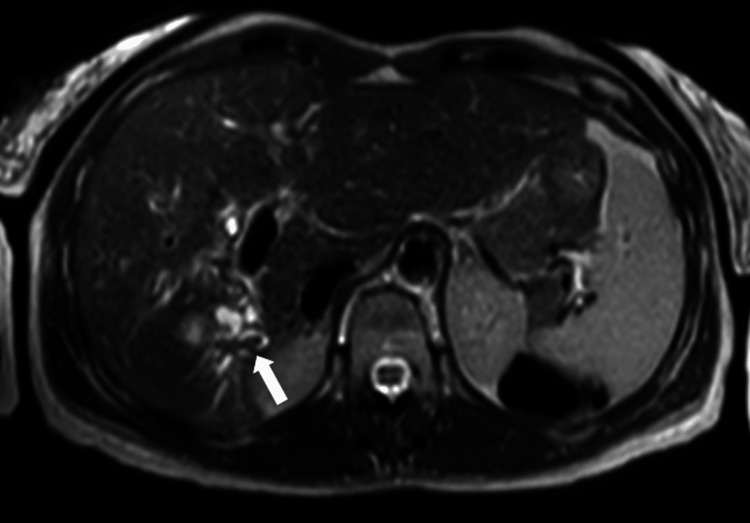
Axial T2-weighted MRI image of the liver Axial T2-weighted MRI showing biliary casts that appear isotense to hypointense relative to bile (white arrow).

**Figure 10:**
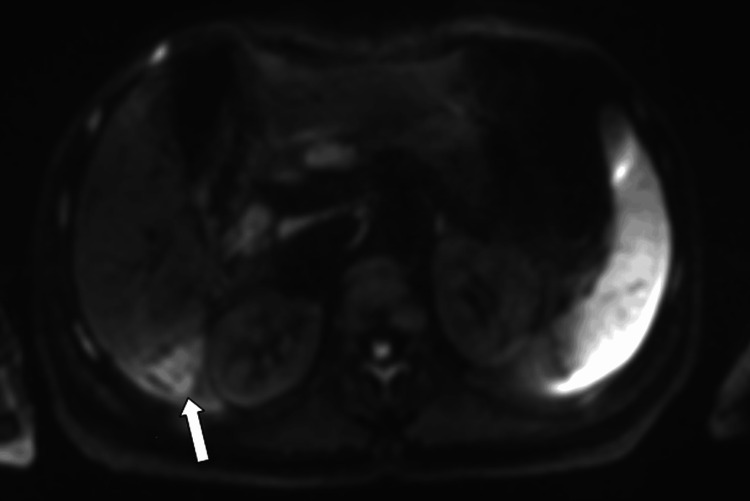
Axial diffusion-weighted MRI (b-value 800) image at the level of the lesion Axial diffusion-weighted MRI (b-value 800) demonstrating hyperintensity of the intraductal lesion in segment VI, consistent with restricted diffusion (white arrow).

**Figure 11:**
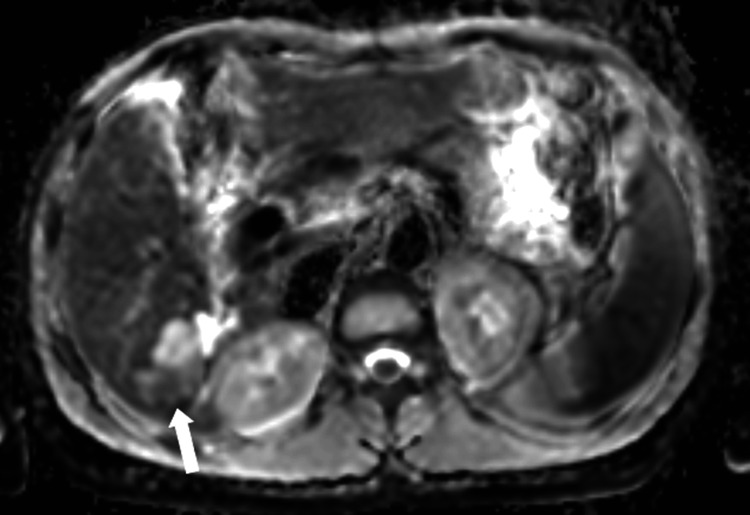
Axial apparent diffusion coefficient map at the level of the lesion Corresponding apparent diffusion coefficient map showing low signal within the lesion, confirming diffusion restriction in the solid intraductal component (white arrow).

**Figure 12:**
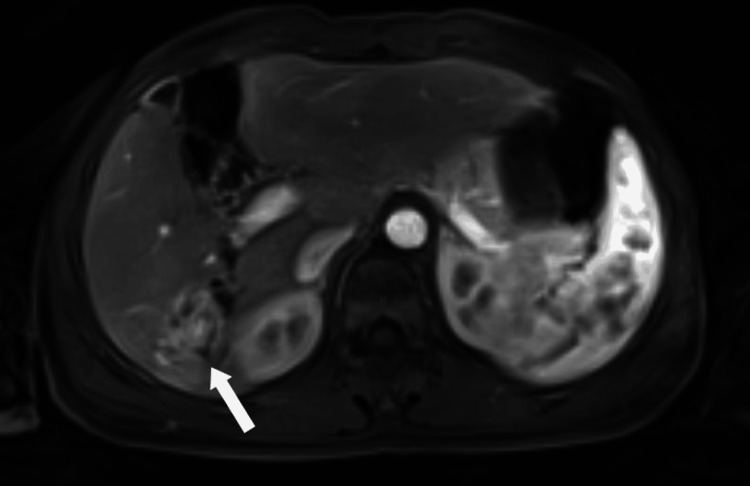
Axial contrast-enhanced MRI in the early arterial phase at the level of the lesion Contrast-enhanced MRI in the early arterial phase showing early enhancement of the intraductal lesion (white arrow).

**Figure 13:**
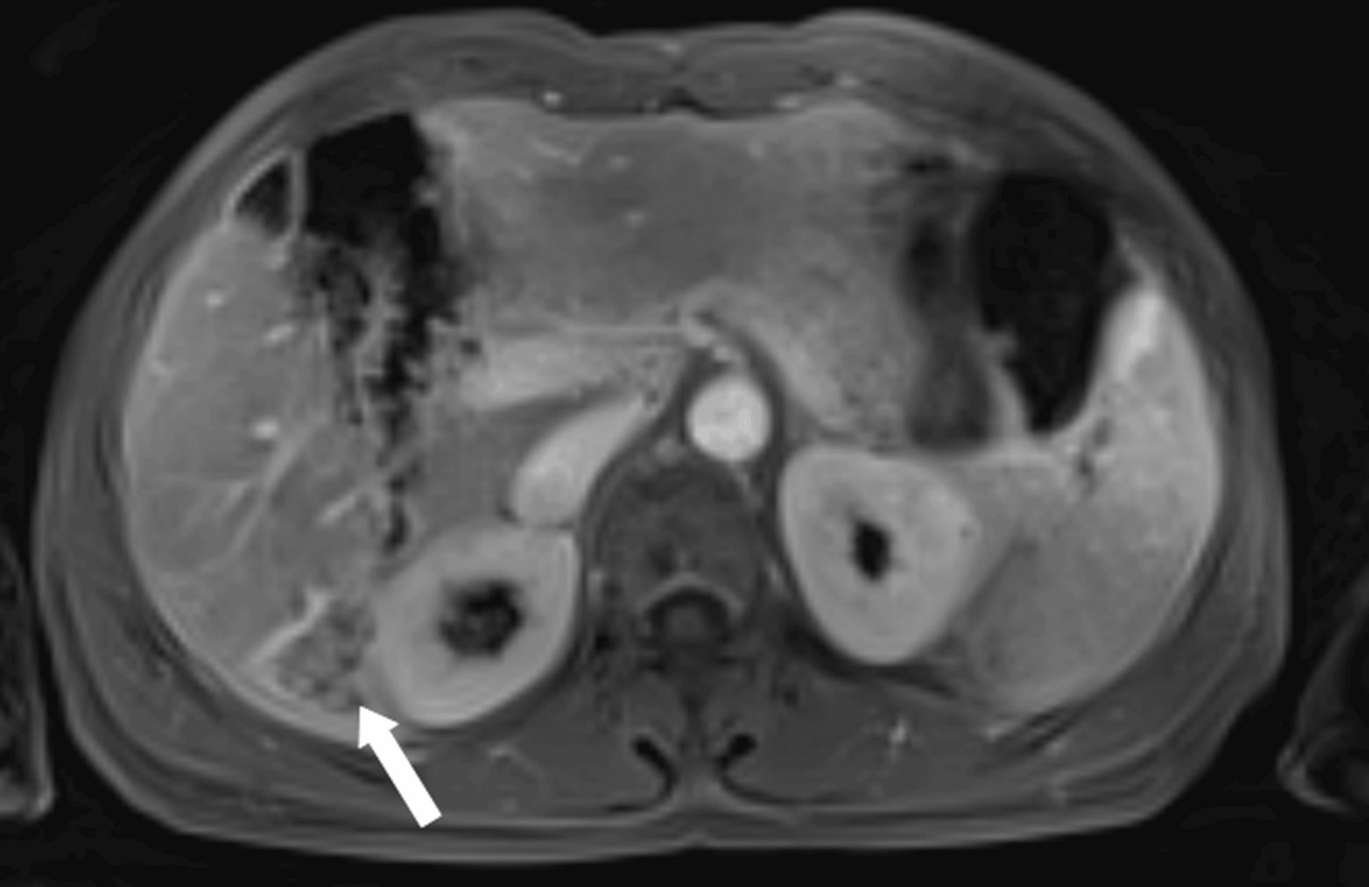
Axial contrast-enhanced MRI in the late venous phase at the level of the lesion Late venous phase MRI demonstrating the lesion becoming isotense to slightly hypointense relative to the liver parenchyma (white arrow).

**Figure 14:**
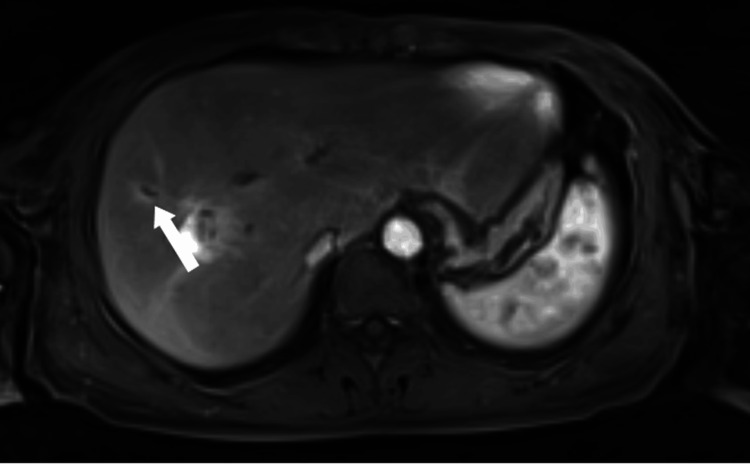
Axial contrast-enhanced MRI of the liver in the early arterial phase Contrast-enhanced MRI showing periductal enhancement in other parts of the liver, suggesting an associated inflammatory biliary process (white arrow).

Biliary casts were identified, appearing hyperintense on T1-weighted images (Figure 8) and isotense to hypointense on T2-weighted images (Figure 9). Diffusion-weighted imaging (Figures 10, 11) demonstrated mild diffusion restriction within the solid component of the lesion, consistent with increased cellularity. Following contrast administration, the lesion showed early arterial phase enhancement (Figure 12) and became isointense to slightly hypointense relative to the liver parenchyma on late venous phase images (Figure 13). Periductal enhancement in other areas of the liver (Figure 14) suggested an underlying inflammatory biliary process.

Histopathological examination of the right hepatic lobe (partial hepatectomy) confirmed a large IPNB of mixed intestinal and biliary type and high grade (grade 3), measuring up to 4.6 cm, corresponding to the intraductal papillary lesion identified on imaging. Within this lesion, two foci of invasive cholangiocarcinoma (maximum diameter 0.5 cm) were identified, confined to the bile duct wall without invasion of the hepatic parenchyma or surrounding adipose tissue. The diffusion restriction observed on MRI correlates with both the increased cellularity of the high-grade lesion and the presence of the invasive components within the bile duct wall. The tumor was completely resected with negative margins (R0 resection). Additionally, a macrometastatic lymph node at the porta hepatis with capsular invasion and extracapsular extension was identified. Background liver parenchyma demonstrated features of PSC with predominantly advanced fibrosis (F3) and focal cirrhosis (F4), correlating with the diffuse beaded appearance of the intrahepatic bile ducts on MRCP. Pathological staging (TNM, 9th edition) was pT1aN1Mx.

## Discussion

IPNB is an uncommon biliary epithelial tumor that is recognized as a precursor lesion of cholangiocarcinoma and follows a dysplasia-carcinoma sequence similar to IPMNs [2]. Because invasive carcinoma is present in a significant proportion of cases at the time of diagnosis, early recognition of this entity is clinically important.

The disease shows a higher prevalence in East Asian regions, where conditions leading to chronic biliary inflammation or biliary stasis -- such as hepatolithiasis and parasitic infections -- are more common. IPNB typically affects patients between 50 and 70 years of age and demonstrates a slight male predominance [4,9]. Because these tumors arise within the bile ducts, clinical symptoms are often related to biliary obstruction or mass effect and may include abdominal discomfort, abdominal pain, anorexia, fever, or jaundice [10].

The radiological appearance of IPNB depends largely on the relative contributions of papillary epithelial proliferation and mucin secretion, as well as the anatomical distribution of the lesion. When papillary tumor growth predominates and mucin production is limited, imaging commonly demonstrates an intraductal mass with upstream biliary dilatation. Conversely, excessive mucin production may lead to diffuse bile duct dilatation without a clearly visible mass. The most frequently observed imaging pattern consists of an intraductal mass associated with both proximal and distal bile duct dilatation due to obstruction of bile flow. Another recognized pattern is focal aneurysmal or cystic dilatation of the bile duct containing an internal papillary component [8]. When a mass is present, it typically appears isotense to hypointense on T1-weighted images and hyperintense on T2-weighted images relative to the hepatic parenchyma. On CT and MRI, enhancement patterns may vary. Although lesions often enhance similarly to the surrounding liver tissue, delayed enhancement may be present, particularly when fibrosis or an invasive component is present. In this case, the absence of marked delayed enhancement likely reflects the predominance of IPNB, while subtle enhancement characteristics may relate to the small invasive foci. Similarly, diffusion restriction is not a defining feature of IPNB itself but is more closely associated with increased cellularity and, importantly, invasive carcinoma. In this case, the observed diffusion restriction most likely corresponds to a high-grade lesion and foci of invasive cholangiocarcinoma within the lesion [11]. The main differential diagnoses include hepatolithiasis, hepatocellular carcinoma with bile duct invasion, and intraductal metastases. Hepatolithiasis typically appears hyperdense on CT and hyperintense on T1-weighted MRI. Hepatocellular carcinoma invading the bile ducts usually presents as a hypervascular hepatic mass demonstrating washout on delayed phases. Intraductal metastases are generally associated with a known primary malignancy. In cases where only bile duct dilatation is present, mucin may be visualized on MRCP as linear hypointense filling defects within the ducts. Endoscopic retrograde cholangiopancreatography may then help localize the lesion within the most dilated ductal segment. The most typical imaging appearance of IPNB remains an intraductal mass with both upstream and downstream biliary dilatation, although a less common cystic variant may appear as a cystic lesion with internal papillary projections communicating with the bile ducts [12].

Another precursor lesion of the biliary tract relevant to this case is biliary intraepithelial neoplasia (BilIN). BilIN is considered the principal precursor lesion of biliary tract carcinoma, particularly the non-papillary form of invasive cholangiocarcinoma [13]. These lesions are frequently associated with chronic inflammatory biliary diseases such as PSC or hepatolithiasis and may occur in both large intrahepatic and extrahepatic bile ducts, as well as in the gallbladder. Macroscopically, BilIN lesions are typically not visible, although subtle mucosal thickening may occasionally be observed. Consequently, they are usually asymptomatic and often detected incidentally [10]. Histologically, BilIN encompasses a spectrum of epithelial alterations, including flat, micropapillary, and proliferative growth patterns [14]. Traditionally, these lesions were graded using a three-tier system (BilIN-1, BilIN-2, and BilIN-3) according to the degree of cytoarchitectural atypia. However, the most recent WHO classification has simplified this system into two categories: low-grade and high-grade dysplasia [15]. In clinical practice, BilIN is most often identified incidentally in patients with underlying hepatobiliary disease or within surgical resection specimens obtained from cholangiocarcinoma.

PSC plays a central role in this case and provides the pathological substrate for biliary carcinogenesis. The MRCP findings of diffuse intrahepatic bile duct dilatation with multifocal strictures, resulting in a characteristic beaded appearance, directly correlate with the histopathological findings of PSC with advanced fibrosis and focal cirrhosis. Chronic biliary inflammation, epithelial injury, and regenerative changes in PSC promote a multistep neoplastic process, facilitating the development of precursor lesions such as BilIN and, less commonly, IPNB, which may arise within large intrahepatic bile ducts in this inflammatory milieu. Although IPNB is less classically associated with PSC than BilIN, increasing evidence suggests that chronic cholestatic injury and bile stasis may contribute to its development [16]. In this context, PSC not only explains the diffuse ductal abnormalities observed on imaging but also represents a key risk factor underlying the coexistence of BilIN, IPNB, and invasive cholangiocarcinoma in this patient, as well as being closely associated with the patient’s underlying condition of ulcerative colitis.

This case illustrates several features that make it particularly noteworthy from both a clinical and radiological perspective. First, the lesion was detected incidentally on CT performed for suspected nephrolithiasis, highlighting how IPNB may initially present as an unexpected finding during imaging for unrelated symptoms. The hepatic lesion appeared relatively non-specific on CT, but the subtle associated intrahepatic bile duct dilatation raised suspicion for a biliary origin and prompted further evaluation. MRI with MRCP played a key role in establishing the correct diagnosis by demonstrating the characteristic appearance of a papillary intraductal lesion within a dilated bile duct. The lesion showed typical imaging features of IPNB, including T2 hyperintensity, T1 hypointensity, diffusion restriction within the solid component, and early contrast enhancement. This case therefore emphasizes the importance of MRI with MRCP in distinguishing intraductal biliary tumors from other hepatic lesions that may appear similar on CT [17].

Another important aspect of this case is the coexistence of multiple biliary neoplastic precursor lesions. Histopathology revealed IPNB with high-grade dysplasia together with two foci of invasive cholangiocarcinoma, as well as BilIN. In addition, the surrounding liver parenchyma demonstrated PSC with advanced fibrosis. The simultaneous presence of PSC, BilIN, IPNB, and invasive carcinoma illustrates the complex spectrum of biliary carcinogenesis and highlights the role of chronic biliary inflammation as an important risk factor. Finally, the case demonstrates how careful radiological evaluation can lead to early recognition of a pre-invasive biliary tumor and guide curative surgical treatment. The identification of an intraductal mass associated with segmental bile duct dilatation should prompt consideration of IPNB, particularly in patients with underlying inflammatory biliary disease.

## Conclusions

IPNB is an uncommon but important precursor lesion of cholangiocarcinoma that can present with variable and sometimes subtle imaging findings. This case highlights the diagnostic value of multimodality imaging, particularly MRI with MRCP, in identifying intraductal biliary tumors. The presence of an intraductal lesion associated with segmental bile duct dilatation should raise suspicion for IPNB. Early recognition is essential, as these lesions frequently coexist with or progress to invasive cholangiocarcinoma. In addition, underlying inflammatory biliary diseases such as PSC may contribute to the development of biliary precursor lesions. Careful radiological evaluation and correlation with clinical context are therefore crucial for timely diagnosis and appropriate surgical management.

## References

[1] Nakanuma Y, Sato Y, Kakuda Y (2024). Interobserver agreement of pathologic classification and grading of tumoral intraductal pre-invasive neoplasms of the bile duct. Ann Diagn Pathol.

[2] Lee MH, Katabathina VS, Lubner MG, Shah HU, Prasad SR, Matkowskyj KA, Pickhardt PJ (2021). Mucin-producing cystic hepatobiliary neoplasms: updated nomenclature and clinical, pathologic, and imaging features. Radiographics.

[3] Adsay V, Mino-Kenudson M, Furukawa T (2016). Pathologic evaluation and reporting of intraductal papillary mucinous neoplasms of the pancreas and other tumoral intraepithelial neoplasms of pancreatobiliary tract: recommendations of Verona consensus meeting. Ann Surg.

[4] Ohtsuka M, Shimizu H, Kato A (2014). Intraductal papillary neoplasms of the bile duct. Int J Hepatol.

[5] Mocchegiani F, Vincenzi P, Conte G, Nicolini D, Rossi R, Cacciaguerra AB, Vivarelli M (2023). Intraductal papillary neoplasm of the bile duct: the new frontier of biliary pathology. World J Gastroenterol.

[6] Toti L, Manzia TM, Di Giuliano F (2024). Intraductal papillary neoplasms of the bile duct: clinical case insights and literature review. Clin Pract.

[7] Nakanuma Y, Uesaka K, Okamura Y (2021). Reappraisal of pathological features of intraductal papillary neoplasm of bile duct with respect to the type 1 and 2 subclassifications. Hum Pathol.

[8] Dias NG, Mendes JT, Kozlowski BA (2025). Intraductal papillary neoplasm of the bile duct: simplifying findings. Semin Ultrasound CT MR.

[9] Kim JR, Jang KT, Jang JY (2023). Intraductal papillary neoplasm of the bile duct: review of updated clinicopathological and imaging characteristics. Br J Surg.

[10] Geramizadeh B (2020). Precursor lesions of cholangiocarcinoma: a clinicopathologic review. Clin Pathol.

[11] Jain K (2023). Intraductal papillary neoplasm of the bile duct: radiological diagnosis of a rare entity: case series. Euroasian J Hepatogastroenterol.

[12] Park HJ, Kim SY, Kim HJ (2018). Intraductal papillary neoplasm of the bile duct: clinical, imaging, and pathologic features. AJR Am J Roentgenol.

[13] Vij M, Puri Y, Rammohan A, G G, Rajalingam R, Kaliamoorthy I, Rela M (2022). Pathological, molecular, and clinical characteristics of cholangiocarcinoma: a comprehensive review. World J Gastrointest Oncol.

[14] Nakanuma Y, Kakuda Y, Sugino T, Sato Y, Fukumura Y (2022). Pathologies of precursor lesions of biliary tract carcinoma. Cancers (Basel).

[15] Chung T, Park YN (2022). Up-to-date pathologic classification and molecular characteristics of intrahepatic cholangiocarcinoma. Front Med.

[16] Ghenea CS, Mihailia M, Negoiţă LM (2024). Review of a rare precancerous lesion - intraductal papillary neoplasm of the bile duct (IPNB). JMRO.

[17] Al-Dhuhli H (2009). Role of magnetic resonance cholangiopancreatography in the evaluation of biliary disease. Sultan Qaboos Univ Med J.

